# The integrin-binding defective FGF2 mutants potently suppress FGF2 signalling and angiogenesis

**DOI:** 10.1042/BSR20170173

**Published:** 2017-04-10

**Authors:** Seiji Mori, Nobuaki Hatori, Naomasa Kawaguchi, Yoshinosuke Hamada, Tsung-Chieh Shih, Chun-Yi Wu, Kit S. Lam, Nariaki Matsuura, Hirofumi Yamamoto, Yoko K. Takada, Yoshikazu Takada

**Affiliations:** 1 Department of Medical Technology, Faculty of Health Sciences, Morinomiya University of Medical Sciences, 1-26-16 Nanko-kita, Suminoe, Osaka 559-8611, Japan; 2 Department of Molecular Pathology, Division of Health Sciences, Osaka University Graduate School of Medicine, 1-7 Yamadaoka, Suita, Osaka 565-0871, Japan; 3 Department of Biochemistry and Molecular Medicine, School of Medicine, University of California Davis, Sacramento, California 95817, United States of America; 4 Department of Dermatology, School of Medicine, University of California Davis, Sacramento, California 95817, United States of America; 5 The PhD Program for Translational Medicine, College of Medical Science and Technology, Taipei Medical University, 250 Wu-Hsing Street, Taipei 11031, Taiwan, R.O.C

**Keywords:** angiogenesis, antagonists, fibroblast growth factors, integrins

## Abstract

We recently found that integrin αvβ3 binds to fibroblast growth factor (FGF)-αvβ31 (FGF1), and that the integrin-binding defective FGF1 mutant (Arg-50 to glutamic acid, R50E) is defective in signalling and antagonistic to FGF1 signalling. R50E suppressed angiogenesis and tumour growth, suggesting that R50E has potential as a therapeutic. However, FGF1 is unstable, and we had to express R50E in cancer cells for xenograft study, since injected R50E may rapidly disappear from circulation. We studied if we can develop antagonist of more stable FGF2. FGF2 is widely involved in important biological processes such as stem cell proliferation and angiogenesis. Previous studies found that FGF2 bound to αvβ3 and antagonists to αvβ3 suppressed FGF2-induced angiogenesis. However, it is unclear how FGF2 interacts with integrins. Here, we describe that substituting Lys-119/Arg-120 and Lys-125 residues in the predicted integrin-binding interface of FGF2 to glutamic acid (the K119E/R120E and K125E mutations) effectively reduced integrin binding to FGF2. These FGF2 mutants were defective in signalling functions (ERK1/2 activation and DNA synthesis) in NIH3T3 cells. Notably they suppressed, FGF2 signalling induced by WT FGF2 in endothelial cells, suggesting that the FGF2 mutants are antagonists. The FGF2 mutants effectively suppressed tube formation *in vitro*, sprouting in aorta ring assays *ex vivo* and angiogenesis *in vivo*. The positions of amino acids critical for integrin binding are different between FGF1 and FGF2, suggesting that they do not interact with integrins in the same manner. The newly developed FGF2 mutants have potential as anti-angiogenic agents and useful tools for studying the role of integrins in FGF2 signalling.

## Introduction

The fibroblast growth factor (FGF) family consists of 22 related polypeptides that are expressed in almost all tissues and are multifunctional. Some FGFs, like FGF1 and FGF2, have potent angiogenic activity and are implicated as promoters of angiogenesis, the formation of new blood vessels, in cancer and chronic inflammatory diseases [1–3]. FGFs also increase the motility and invasiveness of a variety of cell types. The biological effects of FGFs are mediated by four structurally related receptor tyrosine kinases: FGF receptor (FGFR)-1 (FGFR1), FGFR2, FGFR3 and FGFR4. The binding of FGF to its receptor results in receptor dimerization and subsequent autophosphorylation of specific tyrosine residues within the intracellular domain. This leads to the activation of intracellular signalling cascades. Integrins are a family of cell adhesion receptors that recognize extracellular matrix (ECM) ligands and cell surface ligands [4]. Integrins are transmembrane α−β heterodimers, and at least 18 α and 8 β subunits are known [5]. Integrins are involved in signal transduction upon ligand binding and their functions are in turn regulated by signals from within the cell [5]. Previous studies found that antagonists to integrin αvβ3 suppressed angiogenesis induced by FGF2 [6], suggesting that this integrin is involved in FGF2 signalling (FGF–integrin cross-talk). Cross-talk between integrins and FGFs are an important signalling mechanism during normal development and pathological processes [7]. Current models of integrin-growth factor cross-talk propose that integrins contribute to growth factor signalling through interaction of integrins with the ECM [8–10]. We previously reported that FGF1 specifically binds to the classical RGD-binding site of integrin αvβ3 (KD approximately 1 μM) using docking simulation and mutagenesis [11,12]. The integrin-binding site in FGF1 is distinct from the FGFR-binding site. An FGF1 mutant (Arg-50 to glutamic acid mutant, R50E), which is defective in integrin binding but still binds to heparin and FGFR1, is defective in inducing DNA synthesis, cell proliferation, cell migration and chemotaxis, suggesting that the direct integrin binding to FGF1 is critical for FGF1 signalling [13]. We propose a model in which integrin and FGFR bind to FGF1 simultaneously and make a ternary complex on the cell surface. We discovered that R50E is a dominant-negative antagonist of FGF1. R50E suppressed DNA synthesis and cell proliferation induced by WT FGF1 [13], and suppressed angiogenesis *in vitro* and *in vivo* [14]. Using cancer cells that stably express WT FGF1 or R50E, we showed that WT FGF1 markedly enhanced tumour growth and R50E suppressed it [14]. Therefore, the R50E mutant of FGF1 has potential as a therapeutic (FGF1 decoy). FGF1 is, however, thermodynamically unstable (*T*_m_ =40°C). We had to express R50E in cancer cells to demonstrate its antagonistic effects on tumour growth and angiogenesis, since, we believed that R50E is quickly removed from circulation if we inject R50E systematically into mice in xenograft experiments [14]. FGF2 is thermodynamically more stable (*T*_m_ =59°C) and has a longer half-life in circulation than FGF1. FGF2 is widely involved in important biological processes such as stem cell proliferation and angiogenesis partly due to its stability [1]. It has been reported that integrin αvβ3 binds to immobilized FGF2 and promotes endothelial cell adhesion and spreading [15]. Also, anti-αvβ3 monoclonal and polyclonal antibodies specifically inhibit cell proliferation and up-regulation of the urokinase type plasminogen activator induced by soluble FGF2 in GM 7373 cells grown on tissue culture plastic [15]. It is unclear, however, how FGF2 interacts with integrins. The goal of the present study is to determine how integrins bind to FGF2 and to determine if integrin-binding defective FGF2 is dominant negative as in the case of FGF1. In the present study, we have developed FGF2 mutants that are defective in integrin binding and found that such FGF2 mutants act as antagonists of FGF2 signalling (FGF2 decoys). Notably, these FGF2 mutants effectively suppressed angiogenesis. Such FGF2 mutants have potential as therapeutics.

## Materials and methods

### Materials

All chemicals were purchased from Thermo Fisher Scientific, Sigma (St. Louis, MO) or Nacalai Tesque (Kyoto, Japan) unless otherwise stated. NIH3T3 embryonic mouse fibroblasts were obtained from American Type Culture Collection (A.T.C.C.) and were maintained in DMEM supplemented with 10% FBS and antibiotics. Human umbilical endothelial cells (HUVECs) were purchased from Sanko Junyaku (Tokyo, Japan) and were routinely cultured in EGM-2 Bullet kit (Lonza, Basel, Switzerland) supplemented with 2% FBS. K562 erythroleukaemia cells and K562 cells that express recombinant αvβ3 were described before [11]. Recombinant soluble αvβ3 was synthesized as described [16]. Experiments involving animals have been performed in accordance with the legal requirement of the UC Davis and the Osaka University. Experiments using human subjects have been performed in accordance with the Declaration of Helsinki (2013) of the Worrld Medical Association.

### Synthesis of FGF2

A fragment of cDNA encoding human FGF2 (PALPEDGGSGAFPPGHFKDPKRLYCKNGGFFLRIHPDGRVDGVREKSDPHIKLQLQAEERGVVSIKGVCANRYLAMKEDGRLLASKCVTDECFFFERLESNNYNTYRSRKYTSWYVALKRTGQYKLGSKTGPGQKAILFLPMSAKS) was amplified by PCR using full length human FGF2 cDNA as a template and subcloned into the BamHI/EcoRI site of PET28a+. Protein was synthesized in *Escherichia coli* BL21 and purified by Ni-NTA affinity chromatography. WT and mutant FGF2 migrated as single bands in SDS/PAGE (results not shown).

### Docking simulation

Docking simulation of interaction between FGF2 (PDB code 2FGF) and integrin αvβ3 (PDB code 1L5G, open-headpiece form) was performed using AutoDock 3.05 as described [11]. Cations were not present in αvβ3 during docking simulation [11,16]. The ligand is presently compiled to a maximum size of 1024 atoms. Atomic solvation parameters and fractional volumes were assigned to the protein atoms by using the AddSol utility and grid maps were calculated by using AutoGrid utility in AutoDock 3.05. A grid map with 127 × 127 × 127 points and a grid point spacing of 0.603 Å included the headpiece of αvβ3 (residues 1–438 of αv and residues 55–432 of β3). Kollman ‘united-atom’ charges were used. AutoDock 3.05 uses a Lamarckian genetic algorithm (LGA) that couples a typical Darwinian genetic algorithm for global searching with the Solis and Wets algorithm for local searching. The LGA parameters were defined as follows: the initial population of random individuals had a size of 50 individuals; each docking was terminated with a maximum number of 1 × 10^6^ energy evaluations or a maximum number of 27000 generations, whichever came first; mutation and crossover rates were set at 0.02 and 0.80 respectively. An elitism value of one was applied, which ensured that the top-ranked individual in the population always survived into the next generation. A maximum of 300 iterations per local search were used. The probability of performing a local search on an individual was 0.06, whereas the maximum number of consecutive successes or failures before doubling or halving the search step size was 4.

### Surface plasmon resonance study

Surface plasmon resonance (SPR) was performed as previously described [11]. Briefly, soluble αvβ3 was immobilized on the CM5 sensor chip using a standard amine coupling procedure [17]. The WT and mutant FGF2 were individually two-fold serially diluted from 2 μM in HBS-P buffer (0.01 M Hepes, pH 7.4, 0.15 M NaCl and 0.0005% of surfactant P20) with 1 mM of Mn^2^. Samples were injected at 50 μl/min for 1.8 min. The HBS-P buffer with 1 mM of Mn^2^ was then injected at 50 μl/min for 3 min to allow the bound FGF2s to dissociate from the integrin.

### BrdU incorporation assay

DNA synthesis was measured by the cell proliferation ELISA BrdU kit (Roche Diagnostics, Basel, Switzerland). NIH3T3 cells were starved for 16 h. Cells were stimulated with either WT FGF2 or mutants on 96-well plate for 24 h and concomitantly BrdU solution was added to the culture. We also tested the mixture of WT FGF2 (5 ng/ml) and each mutant (250 ng/m). The amplitude of absorbance at 450 nm is proportional to the BrdU incorporation into the cells.

### Cell migration assay

Cell migration assay was performed as previously described [14]. Briefly, the membrane was placed into a 24-well cell culture plate, and the lower portion of the plate was filled with serum-free EBM-2 medium containing 5 ng/ml WT FGF2, K119E/R120E or K125E. We also tested the mixture of WT FGF2 (5 ng/ml) and individual mutants (5 ng/m) or the mixture. Starved HUVECs (6 × 10^4^ cells/filter) were plated on the filter and incubated at 37°C for 6 h. Chemotaxed cells were stained for visualization and counted.

### Endothelial cell tube formation assay

Endothelial cell tube formation assay was performed as described [14]. In brief, serum-starved HUVECs were plated in wells (3 × 10^4^ cells/well) of 48-well plates coated with 150 μl Matrigel (BD Biosciences, San Jose, CA). The medium contains 5 ng/ml WT FGF2, individual mutants (5 ng/ml), or the mixture (5 and 250 ng/ml respectively). Cells were incubated for 6 h at 37°C. The number of vessel branch points of tube per field was counted from the digital images.

### *Ex vivo* aorta ring assay

Thoracic aortae were isolated from 8-week-old rats and used for aorta ring assay as described previously [14]. Briefly, aortic segments were embedded into type I collagen (Nitta Gelatin, Osaka, Japan) that contains WT FGF2 (50 ng/ml), individual mutants (50 ng/m) or the mixture (50 and 2500 ng/ml respectively). Aortic ring sprouts on day 10 were photographed.

### *In vivo* angiogenesis assay

Hydrogels (MedGEL, Tokyo, Japan) were immersed in WT FGF2 (100 ng/ml), FGF2 mutant (100 ng/ml) or the mixture (100 ng and 5 μg/ml respectively) and were implanted subcutaneously into the back of 10-week-old rats. The epidermis, dermis and subcutaneous tissue were removed 2 weeks after implantation and tissue sections were stained for Von Willebrand factor using antibody specific to Von Willebrand factor (Abcam, Tokyo, Japan) to detect blood vessels. The number of blood vessels was counted under a light microscope.

### Other methods

Mutagenesis of FGF2 was performed as previously described [11]. Cell adhesion assays [18] and ELISA-type integrin binding assays [19] were performed as described. Statistical significance was tested in Prism 6 (GraphPad Software) using ANOVA and Tukey’s multiple-comparison test to control the global type I error.

## Results

### Identification of amino acid residues that is critical for integrin binding

**Figure 1:**
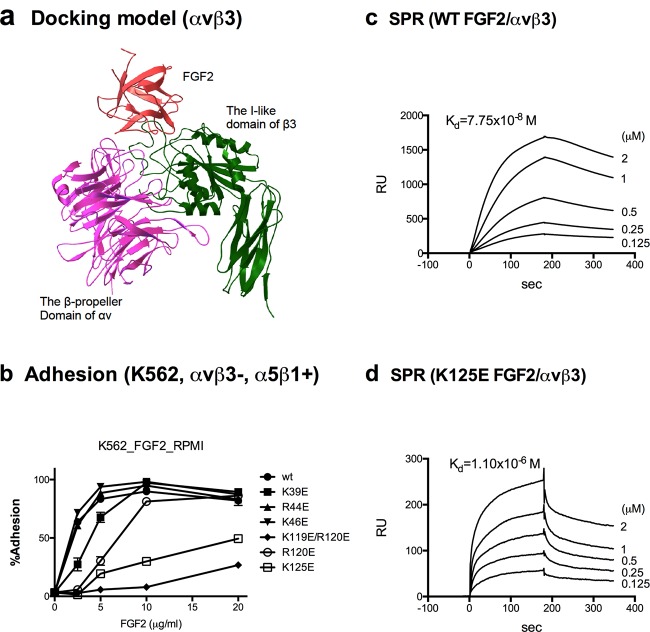
Binding of FGF2 to integrins (**a**) Docking simulation of interaction between FGF2 and integrin αvβ3. Docking simulation was performed as described [11] using the crystal structure of FGF2 (2FGF) and αvβ3 (1LG5). The docking model (docking energy –22.0 kcal/mol) predicts that FGF2 binds to the RGD-binding site of αvβ3 at a high affinity. We chose several amino acid residues of FGF2 (e.g. lysine at position 125, Lys^125^) for mutagenesis studies. (**b**) Binding of FGF2 mutants to α5β1 in adhesion assays. We tested the binding of FGF2 mutants to α5β1 in adhesion assays using K562 erythroleukaemia cells (α5β1+, αvβ3–). K125E and K119E/R120E showed very weak binding. (**c**) Binding of WT FGF2 to soluble αvβ3 in SPR. We fitted on-rate and then off-rate and calculated Kd for individual curves. The Kd value shown is the average of Kd values for individual curves. WT FGF2-bound well to immobilized αvβ3 at *K*_d_ =7.75 × 10^−8^ M, which is consistent with docking simulation and previous reports. (**d**) Binding of K125E FGF2 mutant to soluble αvβ3 in SPR. We fit the curves globally with conformation-change model, in which A + B < = > AB (*K*_d_1) and then AB < = > AB* (*K*_d_2). *K*_d_1 is used as the binding *K*_d_ to compare with WTs *K*_d_. WT K125E bound to immobilized αvβ3 at a low affinity *K*_d_ =1.1 × 10^−6^ M. K119E/R120E did not show detectable binding (not shown).

To identify how integrin αvβ3 and FGF2 interact, we performed docking simulation of interaction between αvβ3 (PDB code 1L5G) and FGF2 (PDB code 2FGF) using autodock3 (Figure 1a). The simulation predicted that FGF2 binds to the classical RGD-binding site of αvβ3 with high affinity (docking energy –22 kcal/mol). When the FGFR1–FGF2 complex (1FQ9) was superposed on the αvβ3–FGF2 complex, there was little or no steric hindrance, suggesting that FGF2 can simultaneously bind to FGFR1 and αvβ3. The predicted integrin-binding interface overlaps with the heparin-binding site (residues 119–128, KRTGQYKLGS). To generate FGF2 mutants that are defective in integrin binding, we introduced mutations into several amino acid residues (Lys^39^, Arg^44^, Lys^46^, Lys^119^/Arg^120^ and Lys^125^ to glutamic acid) within the predicted integrin-binding interface of FGF2 (Table 1). We studied if the FGF2 mutants support adhesion of K562 cells that express recombinant integrin αvβ3 (αvβ3–K562 cells) and parent K562 cells. K562 cells that are deficient in proteoglycans were chosen because FGF2 strongly binds to cell surface proteoglycans (e.g. on CHO cells). Unexpectedly, FGF2 supported adhesion of both αvβ3–K562 and K562 cells to the similar extent, suggesting that FGF2 binds to α5β1 in addition to αvβ3 since α5β1 is the only integrin expressed in K562 cells [20]. We found that the K125 to E mutation (K125E) and K119E/R120E markedly suppressed adhesion of K562 cells to FGF2 (Figure 1b). The R44E mutation, which corresponds to R50E in FGF1, did not affect integrin binding, suggesting that FGF1 and FGF2 do not bind to integrins in the same manner. SPR study using immobilized soluble αvβ3 indicated that WT FGF2 bound to immobilized soluble αvβ3 on the sensor chip at *K*_d_ =7.75 × 10^−8^ M (Figure 1c). The K125E mutant showed lower affinity (*K*_d_ =1.1 × 10^−6^ M) and lower RU than WT FGF2 (*K*_d_ =7.75 × 10^–8^ M) (Figure 1d). The K119E/R120E mutant did not show detectable binding to αvβ3 in SPR. K119E/R120E was defective in binding to heparin, but K125E of FGF2 bound to heparin (results not shown). These findings are consistent with the docking model and K125E and K119E/R120E in the predicted integrin-binding site of FGF2 suppresses FGF2 binding to integrin αvβ3 and α5β1. We do not rule out the possibility, however, that substitution of these residues indirectly affects integrin binding through global conformational changes.

**Table 1:** Amino acid residues of FGF2 and αvβ3 that are in the predicted interface

FGF2	αv	β3
Asn-27, **Arg-39**, Glu-78, Asp-79, Gly-80, Lys-110, Tyr-111, Thr-112, Ser-113, Trp-114, **Lys-119, Arg-120**, Thr-121, Gln-123, Tyr-124, **Lys-125**, Leu-126, Ser-128, Lys-129, Thr-130, Gly-131, Pro-132, Gly-133, Gln-134	Met-118, Gln-145, Asp-146, Ile-147, Asp-148, Ala-149, Asp-150, Gly-151, Phe-177, Tyr-178, Trp-179, Gln-180, Thr-212, Ala-213, Gln-214, Ala-215, Ile-216, Asp-218, Asp-219, Arg-248	Tyr122, Ser-123, Met-124, Lys-125, Asp-126, Asp-127, Asp-179, Met-180, Lys-181, Thr-182, Arg214, Arg-216, Asp-217, Ala-218, Asp-251, Lys-253, Thr-311, Glu-312, Asn-313, Val-314, Ser-334, Met-335

Amino acid residues in integrin αvβ3 and FGF2 within 6 Å to each other in the docking model were identified using Swiss-pdb viewer v. 4.1. Several amino acid residues in FGF2 were selected for mutagenesis (shown in bold).

### K119E/R120E and K125E FGF2 mutants are defective in ERK1/2 activation and in inducing DNA synthesis and suppress DNA synthesis induced by WT FGF2 in NIH3T3 cells

**Figure 2:**
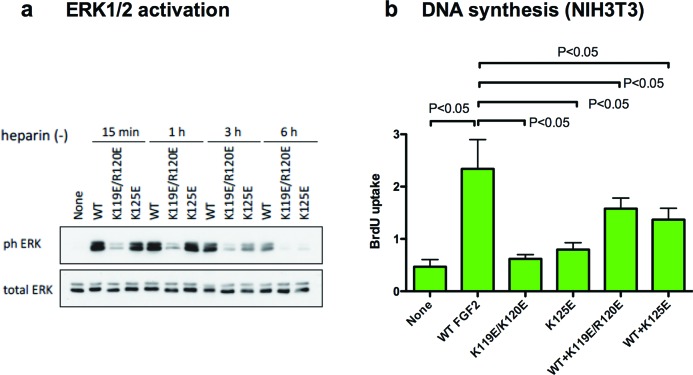
FGF2 mutants are defective in activating Erk1/2 and inducing DNA synthesis and suppressing DNA synthesis induced by WT FGF2 in NIH3T3 mouse embryonic fibroblasts NIH3T3 cells were stimulated with either WT FGF2 (5 ng/ml) or FGF2 mutants (each 5 ng/ml) for indicated periods. Cell lysates were analysed by Western blotting using anti-p-ERK1/2 and total ERK1/2 (**a**). NIH3T3 cells were starved and stimulated with WT FGF2 (5 ng/ml), FGF2 mutants (5 ng/ml) or the mixture of WT FGF2 (5 ng/ml) and mutants (250 ng/m) for 24 hours in the presence of BrdU (**b**). Results are expressed as means ± S.E.M. of the absorbance.

To study the role of integrin binding to FGF2 in FGF2 signalling, we determined if K119E/R120E and K125E induce ERK1/2 phosphorylation in NIH3T3 cells. Sustained ERK1/2 activation (>3 h after stimulation) is integrin dependent and crucial to cell-cycle entry upon FGF stimulation [21,22]. We found that WT FGF2-induced ERK1/2 phosphorylation sustained until 3 h, while K125E-induced ERK1/3 activation diminished after 3 h. K119E/R120E was not able to induce transient or sustained ERK1/2 at all (Figure 2a). These results suggest that K119E/R120E and K125E were both defective in inducing sustained ERK1/2 activation, as in the case of the R50E mutant of FGF1, which induces transient ERK1/2 activation but is defective in sustained ERK1/2 activation [11].

We determined if K119E/R120E and K125E induce DNA synthesis using BrdU incorporation assays in NIH3T3 cells. We found that K119E/R120E and K125E were both defective in inducing DNA synthesis (Figure 2b), consistent with the report that DNA synthesis is related to sustained ERK1/2 activation and the ability of FGF2 to bind to integrins [21,22]. Notably, excess FGF2 mutants suppressed WT FGF2-induced DNA synthesis (Figure 2b), suggesting that K119E/R120E and K125E are dominant negative.

### K119E/R120E and K125E FGF2 mutants are defective in inducing ERK1/2 activation and cell migration in HUVECs and suppress HUVEC migration induced by WT FGF2

**Figure 3:**
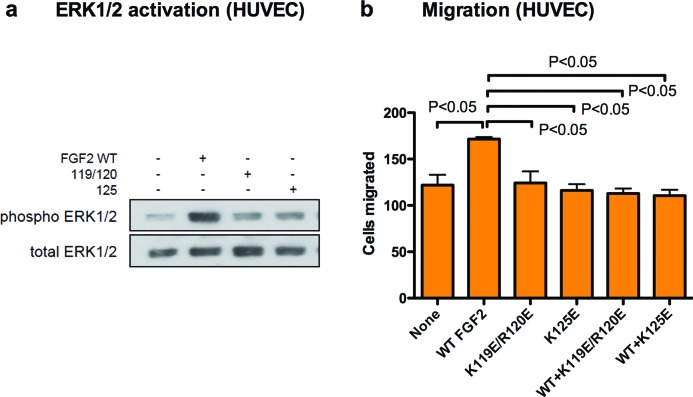
FGF2 mutants are defective in inducing cell migration and suppress cell migration induced by WT FGF2 in HUVECs (**a**) ERK1/2 activation: HUVEC cells were stimulated with WT FGF2 (5 ng/ml) or FGF2 mutants (5 ng/ml) for 60 min. Cell lysates were analysed by Western blotting using anti-p-ERK1/2 and total ERK1/2. (**b**) Cell migration: lower side of the filter in the Transwell chamber was coated with fibronectin (10 μg/ml). The lower chamber was filled with serum-free medium with WT FGF2 (5 ng/ml), mutants (5 ng/ml) or the mixture of WT FGF2 and mutants (5 and 250 ng/ml respectively). HUVECs were plated on the filter and incubated for 6 h. Chemotaxed cells were stained and counted from the digital images. Data are shown as means ± S.E.M. of the number of cells per field.

We determined the ability of K119E/R120E and K125E to suppress FGF2 signalling in HUVECs. We found that WT FGF2-induced ERK1/2 phosphorylation in HUVECs, but K119E/R120E and K125E did not (Figure 3a), suggesting that the FGF2 mutations affect FGF2 signalling in HUVECs as in NIH3T3 cells. Endothelial cell migration is a critical feature of tumour angiogenesis. We examined the effect of the FGF2 mutants on migration of HUVECs. We found that WT FGF2-induced migration of HUVECs, but K119E/R120E or K125E did not (at 5 ng/ml) (Figure 3b). Excess mutants effectively suppressed migration of HUVECs increased by WT FGF2 (Figure 3b). This suggests that K119E/R120E and K125E act as antagonists of FGF2 in migration of HUVECs.

### K119E/R120E and K125E FGF2 mutants are defective in angiogenesis and suppress angiogenesis induced by WT FGF2

We studied if K119E/R120E and K125E FGF2 mutants can induce angiogenesis and if they can suppress angiogenesis induced by WT FGF2 using three different angiogenesis models.

#### In vitro tube formation

**Figure 4:**
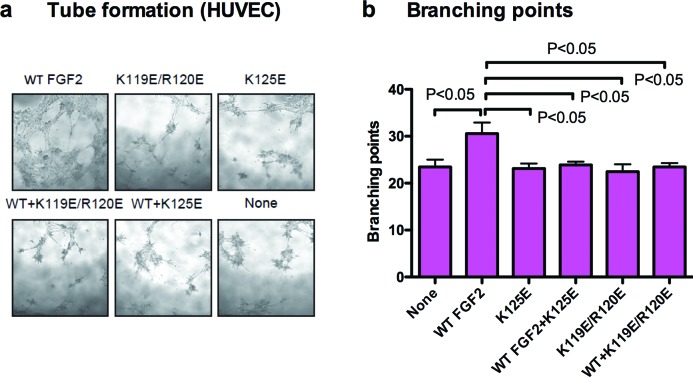
FGF2 mutants are defective in inducing tube formation and suppress tube formation induced by WT FGF2 in HUVECs (**a**) Serum-starved HUVECs were plated on Matrigel-coated plates and incubated with WT FGF2 (5 ng/ml), FGF2 mutants (each 5 ng/ml) or the mixture of WT FGF2 (5 ng/ml) and FGF2 mutants (250 ng/ml) for 8 h. The formation of tube-like structures was observed under bright field. Images of representative tube formation are shown.     (**b**) The number of branching points was counted per field from the images.

We assessed the levels of tube formation by counting the number of branching points in endothelial tube formation assays. Tube formation induced by K119E/R120E and K125E are significantly less than that induced by WT FGF2 (at 5 ng/ml FGF2) (Figure 4a,b). The tube-like structures induced by K119E/R120E and K125E were thin and weak as compared with that induced by WT FGF2 (Figure 4a). Excess K119E/R120E and K125E (at 250 ng/ml) effectively suppressed tube formation induced by WT FGF2 to the background level (Figure 4b). This indicates that K119E/R120E and K125E directly affect endothelial cell tube formation.

#### Ex vivo aorta sprouting assays

**Figure 5:**
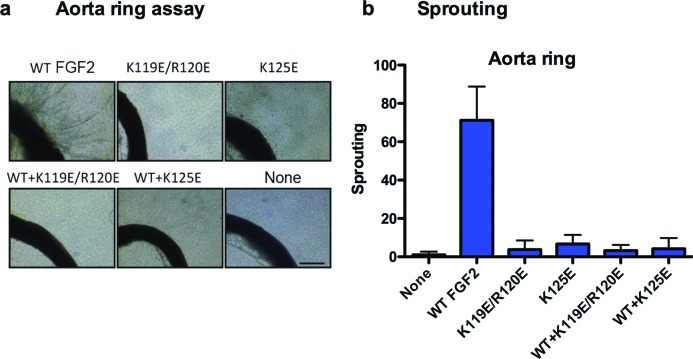
FGF2 mutants suppress WT FGF2-induced vessel sprouting from aorta ring (**a**) Isolated rat aortic ring was embedded in collagen gels in DMEM containing FGF2 WT (50 ng/ml) or mutants (each 50 ng/ml) or the mixture of WT FGF2 (50 ng/ml) and mutants (each 2.5 μg/ml) and cultured for 10 days. Representative images of three independent experiments are shown. (**b**) The areas of sprouting were counted per field from the images. Scale bar =200 μm. Data are shown as means ± S.E.M.

Isolated rat thoracic aortic ring was embedded in collagen gels in DMEM containing WT FGF2, K119E/R120E, K125E or the mixture of WT FGF2 and excess mutants. WT FGF2 (50 ng/ml) markedly induced the sprouting vessels from aortic arch, but the FGF2 mutants (50 ng/ml) did not (Figure 5). Excess K119E/R120E and K125E suppressed the sprouting induced by WT FGF2 to the background levels, suggesting that K119E/R120E and K125E are potent antagonists to FGF2-induced *ex vivo* angiogenesis.

#### In vivo angiogenesis assays

**Figure 6:**
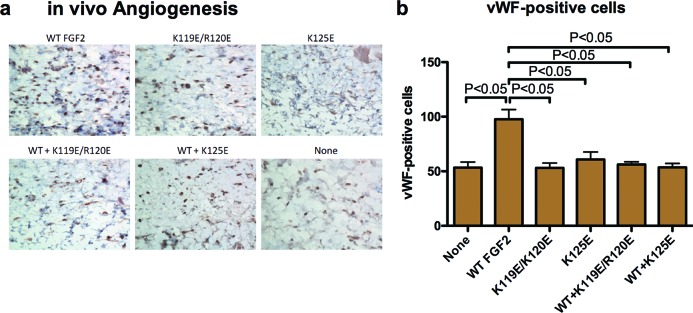
FGF2 mutants suppress WT FGF2-induced angiogenesis (**a**) Hydrogel containing WT FGF2 (100 ng/ml), FGF2 mutants (100 ng/ml) or the mixture of WT FGF2 (100 ng/ml) and excess FGF2 mutants (5 μg/ml) were implanted subcutaneously into the back of rat respectively. The epidermis, dermis and subcutaneous tissue were removed 2 weeks after implantation and tissue sections were stained for Von Willebrand factor. Representative images are shown. (**b**) Three samples were obtained from each condition. Von Willebrand factor positive cells were counted. Data are shown as means ± S.E.M.

We implanted hydrogels that contained WT FGF2, FGF2 mutants or the mixture of WT FGF2 and excess FGF2 mutants subcutaneously into the back of rat. We counted the Von Willebrand factor-positive cells in tissue section to detect blood vessels. We found that WT FGF2 markedly increased the number of blood vessels, whereas FGF2 mutants were defective in this function (Figure 6). Excess FGF2 mutants reduced the number of blood vessel formation induced by WT FGF2. These findings suggest that K119E/R120E and K125E are antagonists of *in vivo* angiogenesis induced by WT FGF2.

Taken together, these results suggest that K119E/R120E and K125E FGF2 mutants are potent anti-angiogenic agents in three different angiogenesis assays.

## Discussion

The present study establishes that substituting Lys-119/Arg-120 and Lys-125 of FGF2 to glutamic acid effectively reduced the binding of FGF2 to integrins. The positions of key residues for integrin binding in FGF2 are not identical with that in FGF1 (Arg-50), suggesting that FGF2 and FGF1 interact with integrins differently, while the structures of FGF1 and FGF2 are superimposable, and the direct binding to integrins is equally critical for both FGF1 and FGF2. It has previously been proposed that the Asp–Gly–Arg (DGR) motif (residues 37–39) in FGF2 is involved in integrin binding [15]. The DGR motif is close to or within the predicted integrin-binding interface of FGF2, but the R39E mutation did not affect integrin binding. This suggests that the DGR motif may not be important for integrin binding. Importantly, the integrin-binding defective FGF2 mutants (K119E/R120E and K125E) are defective in signalling functions (DNA synthesis, sustained ERK1/2 activation and cell migration). Furthermore, they are dominant-negative antagonists of FGF2 signalling and potently suppressed WT FGF2-induced DNA synthesis, ERK1/2 activation and cell migration. These results suggest that the direct binding of FGF2 to integrins is involved in FGF2 signalling, as in the case of FGF1. We have introduced the K119E and R120E mutations individually to FGF2, but these mutations only partially reduced integrin binding and did not affect FGF2 signalling (results not shown). Therefore we need to substitute both Lys^119^ and Arg^120^ to glutamic acid simultaneously to reduce FGF2 signalling.

Notably, K119E/R120E and K125E FGF2 potently suppressed WT FGF2-induced angiogenesis *in vitro* (tube formation), *ex vivo* (aorta ring assays) and angiogenesis *in* *vivo*. These findings suggest that they are potent anti-angiogenic agents. K119E/R120E and K125E are comparable as inhibitors of angiogenesis. It has been proposed that the thermal stability of FGF2 is a major reason why FGF2 is involved in so many more biological processes than FGF1 and why FGF2 is a determinant factor in regulating self-renewal, differentiation, reprogramming in human pluripotent stem cells [23]. The consequence of high stability of FGF2 is a long half-life *in vivo* (7.6 h) [3]. Since FGF2 binds to all FGFR isoforms [24], it is likely that K119E/R120E and K125E FGF2 mutants will be theoretically able to suppress signalling from all members of the FGF family as in the case of FGF1. Therefore, it is likely that K119E/R120E and K125E FGF2 mutants are potentially useful to suppress biological or pathological events that include FGF signalling. Since K125E bound to heparin and K119E/R120E did not, it is likely that the ability to bind heparin may not be directly related to their dominant-negative actions.

Potential advantage of the FGF2 mutants is that: (i) they are highly specific to FGFR compared with tyrosine kinase inhibitors, which are selective rather than specific. Also, (ii) FGF2 mutants are small in size compared with IgG, and therefore FGF2 mutants have better penetrance (access to) to diseased tissues. (iii) Currently used targeting therapeutics (antibodies and kinase inhibitors) almost always induce resistance. This is partly due to point mutations in antibody epitopes or inhibitor-binding sites. Cancer cells obviously benefit from mutations that block the binding of antagonists. We believe that K119E/R120E and K125E of FGF2 may not induce such mutations in FGFR because the FGF2 mutants bind to FGFR exactly the same way as WT FGF2 and block binding of FGF2 (and other members of the FGF family) to FGFR would not benefit cancer cells.

The specifics of the role of integrins in FGF signalling are unclear. αvβ3 integrin is highly expressed in endothelium during angiogenesis and is involved in neovascularization induced by FGF2 [25,26]. Previous studies found that the binding of FGF2 to FGFR is not sufficient to induce cell proliferation in endothelial cells and that an interaction of FGF2 with a cell surface integrin receptor is also required [15]. This is consistent with the observation that anti-αvβ3 antibody inhibited the angiogenic activity exerted *in vivo* by FGF2 without affecting neovascularization induced by vascular endothelial cell growth factor, transforming growth factor-α or phorbol ester [26]. The present study also suggests that integrins other than αvβ3 may bind to FGF2. If this is the case, then, we need to use antagonists to multiple integrins to block FGF2–integrin interaction. It would be necessary to address the integrin-binding specificity of FGF2 in details in future studies. On the other hand, it is likely that the K119E/R120E and K125E mutations suppress binding to multiple integrins regardless of integrin species. If this is the case, then the FGF2 mutants may be more effective than antagonists for individual integrins. The integrin-binding defective FGF2 mutants will be useful to study the molecular details of the FGF2/FGFR/integrin cross-talk.

It is interesting to study if other members of the FGF family require integrin binding for their signalling functions in future studies. If this is the case, then, we may be able to generate dominant-negative inhibitors of FGFs by generating integrin-binding defective mutants.

## Abbreviations

CHO: Chinese hamster ovary
ECM: extracellular matrix
FGF: fibroblast growth factor
FGFR: fibroblast growth factor receptor
HUVEC: human umbilical endothelial cell
LGA: Lamarckian genetic algorithm
SPR: surface plasmon resonance
